# Incidence of cancer among licenced commercial pilots flying North Atlantic routes

**DOI:** 10.1186/s12940-017-0295-4

**Published:** 2017-08-16

**Authors:** Eva Maria Gudmundsdottir, Jon Hrafnkelsson, Vilhjalmur Rafnsson

**Affiliations:** 1 Faculty of Medicine, University of Iceland, Reykjavik, Iceland; 2 Department of Oncology, Landspitali University Hospital, Reykjavik, Iceland; 3 Department of Preventive Medicine, Faculty of Medicine, University of Iceland, Reykjavik, Iceland

**Keywords:** Basal cell carcinoma of skin, Malignant melanoma, Prostate cancer, Cosmic radiation, Aircrew, Cancer registry

## Abstract

**Background:**

To evaluate cancer incidence among licenced commercial pilots in association with cosmic radiation.

**Methods:**

Cohort study where ionizing radiation dose of cosmic radiation was estimated from airline data and software program and cancer incidence was obtained by record linkage with nation-wide cancer registry. All licenced commercial male airline pilots were followed from 1955 to 2015, ever or never employed at airline with international routes. Standardized incidence ratios were calculated and relative risk by Poisson regression, to examine exposure-response relation.

**Results:**

Eighty three cancers were registered compared with 92 expected; standardized incidence ratios were 0.90 (95% CI 0.71 to 1.11) for all cancers, 3.31 (95% CI 1.33 to 6.81) for malignant melanoma, and 2.49 (95% CI 1.69 to 3.54), for basal cell carcinoma of skin. The risk for all cancers, malignant melanoma, prostate cancer, basal cell carcinoma of skin, and basal cell carcinoma of trunk increased with an increase in number of employment years, cumulative air hours, total cumulative radiation dose, and cumulative radiation dose sustained up to age of 40 years. The relative risk for the highest exposure categories of cumulative radiation dose were 2.42 (95% CI 1.50 to 3.92) for all cancers, 2.57 (95% CI 1.18 to 5.56) for prostate cancer, 9.88 (95% CI 1.57 to 190.78) for malignant melanoma, 3.61 (95% CI 1.64 to 8.48) for all basal cell carcinoma, and 6.65 (95% CI 1.61 to 44.64) for basal cell carcinoma of trunk.

**Conclusions:**

This study was underpowered to study brain cancer and leukaemia risk. Basal cell carcinoma of skin is radiation-related cancer, and may be attributed to cosmic radiation. Further studies are needed to clarify the risk of cancers in association with cosmic radiation, other workplace exposure, host factors, and leisure sun-exposure, as clothes, and glass in cockpit windows shield pilots from the most potent ultraviolet-radiation.

## Background

Previous cancer incidence studies on airline pilots have reported increased incidence of specific cancers, predominantly skin cancer [1, 2], prostate cancer [1, 3], and brain cancer [3]. In some studies there are indications of increased risk of the radiation-related cancer, acute myeloid leukaemia [3, 4]; however, that result has not been confirmed in a larger study [1]. In studying the occupational exposure of pilots, attention has been drawn to ionizing radiation of cosmic origin [5]. Cosmic radiation at the cruising altitude of commercial jet flights is a mixture of primary and secondary radiation, mainly gamma rays and neutrons and other particles, including heavy nuclei [6], where the neutrons constitute 30 to 60% of the radiation.

Studies on flight attendants have also reported increased incidence of cancers, both skin and breast cancer [7–9].

As early as 1999, the International Agency for Research on Cancer (IARC) concluded that there was sufficient evidence that neutrons are carcinogenic to humans [10]; however, the conclusion was based on results from animal studies only, as adequate studies on humans were not available. Other potential exposures of pilots are often divided into occupational and leisure time/lifestyle factors. Mentioned in their working environment are electromagnetic fields, ozone, noise, exhaust gases from the engine, and circadian rhythm disruption [11]. Lifestyle factors related to social class have been discussed [1], and leisure time sunbathing habits (surrogate of exposure to ultraviolet radiation), host related risk factors for skin cancers have been investigated [12].

The Icelandic cohort of pilots [2], consisting of licenced pilots, and access to airline files, population registry, and nation-wide cancer registry leverage an optimal setting to augment the sizes of the cohort, extend the follow-up time, and accurately estimate the ionizing radiation exposure. The aim was to evaluate incidence of cancer among pilots in association with cosmic radiation.

## Methods

### Cohort and follow-up

The cohort comprised 551 Icelandic male licenced commercial pilots. The primary sources of data were the files of the Icelandic Aviation Authority and the airline Icelandair. This information made it possible to divide the pilots into those ever employed at Icelandair (*n* = 286), and those never employed at Icelandair (other pilots, *n* = 265), and moreover included their personal identification number, date of birth, and date of licence. In addition, we obtained from the airline files (including seniority lists) information on date of first employment, date of termination of employment, and block hours as well as type of aircraft flown, on an annual basis. The block hours were converted to air hour, as explained later. Other pilots may have worked as commercial pilots at other airlines operating on domestic routes or in the coast guard, however their possible flight career is not documented.

Record linkage was performed with the National Registry at Statistics Iceland, and the Icelandic Cancer Registry by using personal identification numbers. Results from record linkage were returned with encrypted identification numbers. Statistics Iceland provided information on eventual dates of emigration and death. The Icelandic Cancer Registry provided information on cancer site, cancer morphology, and date of diagnosis. The Icelandic Cancer Registry started in 1955. Since 1981, incidence of first basal cell carcinoma of the skin (BCC) has been collected separately; therefore all cancers do not include BCC.

### Exposure assessment

Icelandair pilots have flown commercial flights on domestic and international routes. Icelandair has had regular European routes since 1945 and regular transatlantic routes since 1952. Since 1971 they have operated jets on all international routes [2]. The flight routes have used Keflavik (Reykjavik) in Iceland as their hub. The most common destinations in Europe have been Copenhagen, London, Glasgow, Oslo, Stockholm, Hamburg, Luxembourg, and Frankfurt, and the most common destinations in North America have been New York, Boston, Baltimore, Chicago, Minneapolis, Orlando, and Halifax. Almost every flight to Europe took off quite early in the morning, returning to Keflavik in the afternoon with the same crew. Flights to North America usually departed in the afternoon, arriving on the east coast destinations in five to seven hours. This is too long to allow the same crew to make a same-day return flight, so the crew had to layover for one or more nights. All routes since 1958 according to Icelandair timetables were entered into the database. Timetables for each calendar year were divided between winter and summer season, and into domestic and international flights. Timetables included flight code, place of departure and arrival, departure time, arrival time, flight duration, aircraft type, number of flights per day of the week, and calendar year.

A panel of active and retired pilots specified the flight profiles for every route, aircraft type, and calendar year. The profiles included information on taxi time at the departure airport, ascending time, height, and duration of the first cruising altitude, height, and duration of the second cruising altitude, height, and duration of the third altitude if appropriate, descending time, and taxi time at the destination airport. Shorter legs and domestic flights had only one cruising altitude. The block hours, the time from departure gate to arrival gate (registered in the files at the airline for every pilot on annual basis), and the information from the flight profiles made it possible to estimate the air hours for the pilots. The air hour is the time from take-off to landing. Based on the timetables and the flight profiles, the effective ionizing radiation dose was calculated per calendar year per aircraft type, per air hour, according to the CARI-6 program [5, 13]. The effective dose, per hour, for each aircraft type per calendar year was calculated based on the timetables whereas different flight profiles were taken into account of the aircrafts. The CARI-6 is based on the transport code LUIN2000, as explained in the publication of O’Brien, 1996 [14]. The CARI-6 computer program is the sixth edition of galactic cosmic radiation dose calculation software, developed by the U.S. Federal Aviation Administration [13]. The program has been shown to be accurate and in accordance with in-flight measurements [15]. CARI-6 calculates the effective dose in Sievert depending on the relevant factors, e.g. different flight profiles of various aircraft types, shortest routes, duration of flights and calendar year taking into accounts the activity of the sun. The dose per air hour per aircraft type per calendar year from CARI-6 program were multiplied by air hours per aircraft type per calendar year of individual Icelandair pilots when estimating the cumulative radiation dose in mSv [13]. The effective dose of radiation presents the stochastic health risk to the whole body, which is the probability of cancer induction and genetic effects, of low levels of ionizing radiation. It takes into account the type of radiation and the nature of each organ or tissue being irradiated, and enables summation of organ doses due to varying levels and types of radiation, to produce an overall calculated effective dose.

Occupational exposures were assessed using four metrics: a) the number of employment years, b) cumulative air hours, c) cumulative effective dose of radiation in milliSievert (mSv) and d) cumulative effective dose of radiation in mSv up until the individual pilot reached the age of 40 years. The rationale of choosing the dose up to 40 years of age is the indication that exposure after this age did not induce BCC among atomic bomb survivors [16], while the risk for BCC increased 11% with each one-year of decrease in age at exposure.

### Statistical analyses

Person-years, for subsequent standardized incidence ratio (SIR) analyses, were calculated for each pilot from the date they received their licence, or 1 January 1955, the starting point of the Icelandic Cancer Registry, whichever came later. The follow-up of person-years stopped on a date when and if a) an individual moved abroad, b) an individual died or c) the study ended (31 October 2015), whichever came first. Expected values were based on person-years at risk in 5-year age categories and the corresponding cancer incidence of the general male population in Iceland according to the Icelandic Cancer Registry. SIR was calculated using observed divided with expected number of events, and 95% confidence interval (CI) was calculated with Byar-approximation [17]. For completeness, all cancer sites with any cancer case are shown. The SIR was calculated of the entire cohort of pilots for all cancers, and selected cancer sites containing any cancer case, and separately for each subgroup of pilots.

Attempts were made to use Cox-regression analyses to compare cancer incidence in subgroups, however the data did not meet the criteria for Cox-analysis. The criteria for comparing groups in Cox-regression analysis is that the proportional hazards of the groups being compared, is constant in time, i.e. that the curves are parallel in time. That was not the case in this study for all cancers, and several cancer sites. The cancer incidences were thus compared in relation to exposure metrics using Poisson regression, expressed in relative risk (RR). We used the generalized linear model, glm2-package in R [18]. In these analyses the other pilots were defined as unexposed and the Icelandair pilots were divided into two categories depending on the magnitude of each of the exposure metrics. The divisions of the number of employment years, cumulative air hours, total cumulative radiation dose in mSv, and cumulative radiation dose in mSv up to the age of 40 years, were chosen at the proximity of the median for the respective metrics. These calculations were adjusted for age, introduced as a continuous variable. When year of licence of the pilots was introduced into the model as a continuous variable it gave nearly the same results and therefore it was omitted from the analyses.

Nonparametric tests for linear trends in proportions were made in order to calculate p-trend estimates for exposure-response analysis for the various exposure metrics.

Internal analysis among Icelandair pilots only was also made. However, due to a limited number of events in the exposure categories and small groups, no clear association between exposure and the risk of cancers was observed, indicating lack of statistical power, and consequently these results are not shown.

Statistics were performed in R 3.2.2 (Core Team. R: A language and environment for statistical computing. R Foundation for Statistical Computing, Vienna, Austria. 2015).

## Results

Summary characteristics of the cohort are shown in Table 1. Icelandair pilots were younger on average and consequently got their pilot licence later.

**Table 1:** Summary characteristics

	Icelandair pilots	Other pilots	All pilots
Number	286	265	551
Person-years	8514	8993	17,507
Year of birth, median (range)	1959 (1922–1978)	1946 (1901–1979)	1951
Year of license, median (range)	1987 (1944–2000)	1970 (1935–2004)	1977
Years of employment, median (range)	16 (1–43)	NA	NA
Cumulative air hours, median (range)	7961 (58–29,966)	NA	NA
Cumulative mSv, median (range)	22.55 (0.28–83.20)	NA	NA
Cumulative mSv up to age of 40 years, median (range)	5.80 (0–17.17)	NA	NA

*Abbreviations*: MilliSievert (mSv), and not available (NA)

The SIRs for all cancers and selected cancer sites in the entire cohort are shown in Table 2. The number of cancers registered was 83 among pilots compared with expected number of 92 cancers, yielding a SIR of 0.90 (95% CI 0.71 to 1.11). The SIR for malignant melanoma was 3.31 (95% CI 1.33 to 6.81). Additionally, the SIR for BCC was 2.49 (95% CI 1.69 to 3.54).

**Table 2:** Observed and expected number of cancers, standardized incidence ratios, and confidence intervals among all pilots

Cancer site (ICD-10)	Obs	Exp	SIR	(95% CI)
All (C00-C97, D09-D47^a^)	83	92.49	0.90	0.71 to 1.11
Oesophagus (C15)	2	1.73	1.16	0.13 to 4.18
Stomach (C16)	3	3.96	0.76	0.15 to 2.21
Colon and rectum (C18, C20)	7	9.18	0.76	0.31 to 1.57
Gallbladder (C23)	1	0.10	10.15	0.13 to 56.46
Larynx (C32)	1	0.85	1.17	0.02 to 6.52
Bronchus and lung (C33, C34)	5	10.88	0.46	0.15 to 1.07
Bone (C41)	2	0.09	22.45	2.52 to 81.06
melanoma (C43)	7	2.12	3.31	1.33 to 6.81
Other skin cancer (C44)	4	3.75	1.07	0.29 to 2.73
Penis (C60)	1	0.31	3.18	0.42 to 17.72
Prostate (C61)	28	24.92	1.12	0.75 to 1.62
Kidney (C64)	5	4.22	1.18	0.40 to 2.92
Bladder (C67)	4	6.55	0.61	0.16 to 1.56
Eye (C69)	1	0.25	3.95	0.05 to 21.97
Brain (C71, D33)	4	1.69	2.37	0.64 to 6.06
Thyroid gland (C73)	3	1.41	2.13	0.43 to 6.23
Cancer without specification of site (C80)	3	1.62	1.85	0.37 to 5.40
Mature T/NK-cell lymphomas (C84.6)	1	0.32	3.13	0.04 to 17.43
Chronic lymphocytic leukaemia of B-cell type (C91.1)	1	0.94	1.06	0.01 to 5.91
Acute myeloid leukaemia (C92.0)	1	0.64	1.56	0.02 to 8.69
Not included in all cancers:				
Basal cell carcinoma of the skin (C44)	31	12.44	2.49	1.69 to 3.54

*Abbreviations*: International Statistical Classification of Diseases and Related Health Problems 10th Revision (ICD-10), observed (Obs), expected (Exp), standardized incidence ratio (SIR), and confidence interval (CI)

^a^Related to brain: D09, D30, D35, D41, D32–33, D42–43, D44 and D46–47

The SIR for melanoma was 5.48 (95% CI 2.00 to 11.92) in the group of Icelandair pilots, and for BCC the SIR was 3.54 (95% CI 2.22 to 5.37) but the risk for these cancer sites was not increased in other pilots (Table 3).

**Table 3:** Observed and expected number of cancers, SIR and 95% CI among Icelandair pilots and other pilots

	Icelandair pilots				Other pilots			
Cancer site (ICD-10)	Obs	Exp	SIR	95% CI	Obs	Exp	SIR	95% CI
All (C00-C97, D09-D47^a^)	46	43.23	1.06	0.78 to 1.42	37	49.26	0.75	0.53 to 1.04
Oesophagus (C15)	1	0.78	1.29	0.02 to 7.17	1	0.95	1.05	0.01 to 5.86
Stomach (C16)	1	1.55	0.64	0.01 to 3.58	2	2.40	0.83	0.09 to 3.00
Colon and rectum (C18, C20)	3	4.24	0.71	0.14 to 2.07	4	4.94	0.81	0.22 to 2.07
Gallbladder (C23)	0	0.04	-	0.00 to 90.97	1	0.06	17.18	0.22 to 95.57
Larynx (C32)	0	0.37	-	0.00 to 9.81	1	0.48	2.09	0.03 to 11.61
Bronchus and lung (C33, C34)	2	5.10	0.39	0.04 to 1.42	3	5.78	0.52	0.10 to 1.52
Bone (C41)	1	0.04	24.63	0.32 to 137.00	1	0.05	20.63	0.27 to 114.80
Melanoma (C43)	6	1.10	5.48	2.00 to 11.92	1	1.02	0.98	0.01 to 5.45
Other skin cancer (C44)	4	1.79	2.23	0.60 to 5.71	0	1.96	-	0.00 to 1.87
Penis (C60)	0	0.14	-	0.00 to 25.37	1	0.17	5.90	0.08 to 32.82
Prostate (C61)	15	11.78	1.27	0.71 to 2.10	13	13.14	0.99	0.53 to 1.69
Kidney (C64)	5	1.99	2.51	0.81 to 5.86	0	2.23	-	0.00 to 1.64
Bladder (C67)	0	3.03	-	0.00 to 1.21	4	3.53	1.13	0.31 to 2.90
Eye (C69)	1	0.12	8.27	0.11 to 46.01	0	0.13	-	0.00 to 27.72
Brain (C71, D33)	0	0.82	-	0.00 to 4.48	4	0.87	4.60	1.24 to 11.77
Thyroid gland (C73)	3	0.68	4.44	0.89 to 12.97	0	0.73	-	0.00 to 5.01
Cancer w/o specification of site (C80)	1	0.74	1.35	0.02 to 7.51	2	0.88	2.27	0.25 to 8.19
Mature T/NK-cell lymphomas (C84.6)	1	0.16	6.07	0.08 to 33.77	0	0.15	-	0.00 to 23.74
Chronic lymphocytic leukaemia (C91.1)	1	0.43	2.30	0.03 to 12.80	0	0.51	-	0.00 to 7.25
Acute myeloid leukaemia (C92.0)	1	0.30	3.39	0.04 to 18.84	0	0.35	-	0.00 to 10.63
Not included in all cancers:								
Basal cell carcinoma of the skin (C44)	22	6.21	3.54	2.22 to 5.37	9	6.23	1.44	0.66 to 2.74

*Abbreviations*: International Statistical Classification of Diseases and Related Health Problems 10th Revision (ICD-10), observed (Obs), expected (Exp), standardized incidence ratio (SIR), and confidence interval (CI).

^a^Related to brain: D09, D30, D35, D41, D32–33, D42–43, D44 and D46–47

When comparing cancer incidence of Icelandair pilots with that of other pilots, the RR for all cancers was 2.32 (95% CI 1.43 to 3.78), for melanoma the RR was 9.55 (95% CI 1.50 to 187.39), for prostate the RR was 2.30 (95% CI 1.02 to 5.31), and for BCC the RR was 3.30 (95% CI 1.52 to 7.80) adjusted for age.

Table 4 shows the internal analyses, RR, and 95% CI for all cancers, melanoma and prostate cancer, and Table 5 for all BCC, and BCC of trunk by the exposure metrics. A positive exposure-response relation was observed for all cancers, prostate cancer, malignant melanoma, all BCC, and BCC of trunk. For all cancers and these selected cancer sites, the RRs in the highest category of the exposure metrics compared with unexposed were followed by 95% CI, which did not include unity. Further, the RRs for BCC of trunk were higher than the RRs for all BCC in corresponding exposure categories.

**Table 4:** Age adjusted relative risk for all cancers, prostate cancer and malignant melanoma, by employment years, cumulative air hours, cumulative mSv, and cumulative mSv up to age of 40 years

	All primary cancers (C00-C97)			Prostate cancer (C61)			Malignant melanoma (C43)		
Exposure	No	Yes		No	Yes		No	Yes	
metrics	*n* = 474	*n* = 77	RR (95% CI)	*n* = 524	*n* = 27	RR (95% CI)	*n* = 544	*n* = 7	RR (95% CI)
Employment years									
Unexposed	232	33	1.00 (Referent)	253	12	1.00 (Referent)	264	1	1.00 (Referent)
<15	127	5	1.73 (0.51 to 5.18)	132	0	−	131	1	9.33 (0.22 to 596.01)
15+	115	39	2.34 (1.45 to 3.80)	139	15	2.40 (1.10 to 5.23)	149	5	9.55 (1.49 to 187.47)
p-trend			0.002			0.05			0.02
Cumulative air hours									
Unexposed	232	33	1.00 (Referent)	253	12	1.00 (Referent)	264	1	1.00 (Referent)
<10,000	163	7	1.40 (0.49 to 3.73)	170	0	−	169	1	3.27 (0.09 to 160.46)
10,000+	79	37	2.41 (1.50 to 3.90)	101	15	2.61 (1.22 to 5.60)	111	5	10.29 (1.66 to 197.12)
p-trend			0.0001			0.01			0.004
Cumulative mSv									
Unexposed	232	33	1.00 (Referent)	253	12	1.00 (Referent)	264	1	1.00 (Referent)
<25	145	6	1.36 (0.46 to 3.56)	151	0	−	150	1	5.07 (0.14 to 201.23)
25+	97	38	2.42 (1.50 to 3.92)	120	15	2.57 (1.18 to 5.56)	130	5	9.88 (1.57 to 190.78)
p-trend			0.0005			0.02			0.009
Cumulative mSv up to age of 40 years									
Unexposed	232	33	1.00 (Referent)	253	12	1.00 (Referent)	264	1	1.00 (Referent)
<5	120	5	1.53 (0.47 to 4.20)	125	0	−	125	0	−
5+	122	39	2.37 (1.47 to 3.85)	146	15	2.47 (1.13 to 5.41)	155	6	10.34 (1.23 to 86.61)
p-trend			0.003			0.06			0.005

*Abbreviations*: MilliSievert (mSv), relative risk (RR), confidence interval (CI)

**Table 5:** Age adjusted relative risk for BCC, all and of trunk, by employment years, cumulative air hours, cumulative mSv, and cumulative mSv up to age of 40 years

	Basal cell carcinoma of skin, all (C44)^a^			Basal cell carcinoma of skin, of trunk (C44)^a^		
Exposure	No	Yes		No	Yes	
metrics	*n* = 520	*n* = 31	RR (95% CI)	*n* = 540	*n* = 11	RR (95% CI)
Employment years						
Unexposed	256	9	1.00 (Referent)	263	2	1.00 (Referent)
<15	127	5	2.95 (0.70 to 12.74)	130	2	4.29 (0.35 to 65.08)
15+	137	17	3.34 (1.51 to 7.89)	147	7	6.07 (1.46 to 40.86)
p-trend			0.002			0.01
Cumulative air hours						
Unexposed	232	33	1.00 (Referent)	253	12	1.00 (Referent)
<10,000	163	7	1.40 (0.49 to 3.73)	170	0	−
10,000+	79	37	2.41 (1.50 to 3.90)	101	15	2.61 (1.22 to 5.60)
p-trend			0.0003			0.002
Cumulative mSv						
Unexposed	256	9	1.00 (Referent)	263	2	1.00 (Referent)
<25	146	5	1.92 (0.49 to 7.23)	149	2	2.75 (0.25 to 33.24)
25+	118	17	3.61 (1.64 to 8.48)	128	7	6.65 (1.61 to 44.64)
p-trend			0.0005			0.005
Cumulative mSv up to age of 40 years						
Unexposed	256	9	1.00 (Referent)	263	2	1.00 (Referent)
<5	122	3	1.37 (0.27 to 5.74)	123	2	4.51 (0.40 to 54.67)
5+	142	19	3.62 (1.68 to 8.45)	154	7	6.08 (1.46 to 41.06)
p-trend			0.0006			0.01

*Abbreviations*: MilliSievert (mSv), relative risk (RR), confidence interval (CI)

^a^Not included in all cancers

The body locations of the three main histological types of skin cancers among the two subcategories of pilots are shown in Table 6. Malignant melanoma and BCC on trunk are common among the Icelandair pilots.

**Table 6:** Location of the three main histological types of skin cancers among Icelandair pilots and other pilots

	Malignant	Squamous	Basal cell
Location	melanoma (C43)	cell carcinoma (C44)	carcinoma (C44)
Icelandair pilots			
Total	6	4	22
Trunk	3	1	9
Limbs	2	-	2
Unspecified	-	-	1
Head and neck	1	3	10
Other pilots			
Total	1	-	9
Trunk	-	-	2
Limbs	1	-	-
Unspecified	-	-	-
Head and neck	-	-	7

For all cancers absolute event rates for the two subcategories of pilots gave 0.05 for Icelandair pilots and 0.04 for other pilots, per 10 years; and relative risk reduction yielded −15%.

## Discussion

### Principle findings

The study showed increased risk of malignant melanoma and basal cell carcinoma of the skin among all pilots as compared with the general male population, and this increased risk was even higher among commercial airline pilots, and on the contrary lower among other pilots. In the internal analyses, a positive exposure-response relation was observed for incidence of all cancers, prostate cancer, and malignant melanoma, and the exposure metrics of employment years, cumulative air hours, total cumulative radiation dose, cumulative radiation dose sustained up to the age of 40 years. A strong positive exposure-response relation was also observed for incidence of all BCC, and BCC of trunk, not included in all cancers, with these exposures metrics; i.e. the higher the categories of exposures, the higher the incidence of BCC. For the incidence of these cancers, the risk in the highest exposure category compared with the unexposed was increased and the corresponding 95% CI did not include unity. These increased incidences may be related to exposure to cosmic radiation. The exposure metrics are inevitably associated with each other. For example, annual air hours are used to estimate the cumulative radiation dose. The RRs for all cancers, prostate cancer, melanoma, BCC, and BCC of trunk are highest in the highest exposure categories of cumulative radiation dose, and cumulative radiation dose sustained before the age of 40 years as compared with corresponding categories of duration of employment. The differences are not large; however, the same pattern is to be seen for all cancer types. The pilots in the highest exposure category sustained cumulative radiation dose in the range of 25 to 83 mSv in total, and in the corresponding category the cumulative radiation dose is 5 to 17 mSv up to age of 40 years, and these doses are in addition to the background ionizing radiation exposure. The estimated cumulative cosmic radiation doses, albeit low in light of exposure limits [19], consist in large proportion of neutrons, for which carcinogenicity is unknown for humans in terms of the cancer type involved and the magnitude of relevant exposure [10].

### Comparison with other studies

According to IARC, BCC is one of the cancers caused by ionizing radiation [10], and epidemiological studies have reported increased risk for BCC among radiation exposed uranium miners, radiologic technologist, radiologists, non-cancer patients treated with X-rays, children with cancer treated with X-rays, and atomic bomb survivors, reviewed by Karagas and co-workers in 2006 [20]. Radiation exposure before 20 or 40 years of age is most important for the subsequent risk of BCC [16, 21], which concurs with the high risk for BCC found in association with cosmic radiation up to the age of 40 years in the present study. Reports suggested that radiation effects were strong on skin that was unlikely to be exposed to ultraviolet radiation, such as the trunk [16, 21]. In the present study, the RR for BCC of trunk was considerably higher in the internal analysis than the RR for all BCC. In a previous study, sun exposure and host factors associated with skin cancers were of similar frequency among pilots as among the general population in Iceland [12], and confounding due to these factors is thus unlikely in the present study. Of interest is the anatomically skewed distribution of BCC in the present study, with a high proportion located on the trunk among Icelandair pilots as compared with reports on populations in New Hampshire and Arizona [20]. Increased incidence of BCC was reported among airline pilots from Denmark and Finland in the Nordic study [1]; however, the incidence of BCC was not associated with the cumulative radiation dose in that study. Although these doses had been estimated for all pilots [1], the estimates were not specific for the Danish and Finnish pilots with BCC as an outcome.

In a ten-year old review [22], the causal relation of ionizing radiation to malignant melanoma was considered uncertain and the studies reporting on increased risk included groups such as radiologic technologists, Canadian radiation cohort, cancer patients following radiation therapy, children treated with radiation for cancer, and cancer patients treated only with radiotherapy. Some studies reported melanomas arising in the radiation field. In a study of atomic bomb survivors from 1958 to 1996 there was no significant dose response for melanoma based on 10 cases, and it was recognized that skin cancer is rare among the Asian population, and that may be due to different pigmentation characteristics as compared with Caucasians [16]. Malignant melanoma was the most common cancer type among Australians exposed to computed tomography (CT) scans in childhood [23], and increased incidence risk ratio was reported for the melanomas. Two similarly designed studies on cancer risk after CT in childhood did not report on malignant melanoma as an outcome [24, 25]. Quite a few studies on airline pilots have reported increased incidence of malignant melanoma [1, 3, 26]. In the Nordic study of pilots a positive dose-response was shown in relation to ionizing radiation dose [1], and the relative risk for melanoma was increased more than twofold with a cumulative dose of 3 mSv or higher. Among the Nordic pilots [1] and also the pilots in the present study, the melanoma arises predominantly on the trunk and limbs, which are the same anatomical locations for melanoma in the general male population [27]. No control for possible confounding due to host factors and leisure sun exposure was available in the Nordic study [1]; however these risk factors were later reported of similar frequency among pilots and the general population for the Icelandic setting [12].

Among the Icelandair pilots the SIR for squamous cell carcinoma of skin was 2.23, based on four cases; however, the 95% CI included unity. In the Nordic study, the SIR for other skin cancers was 2.08 (95% CI 1.74 to 2.79) [1].

Increased incidence for prostate cancer has been reported in other previous studies on commercial airline pilots [1, 3]. The increased risk of prostate cancer was only seen among the Nordic pilots aged over 60 years, who had the highest number of working hours in long haul aircraft [1]. In that study it was suggested that jet lag might increase the risk of the hormone-related prostate cancer [1]; however, in the Nordic study, the association of prostate cancer with the radiation dose sustained by these pilots was not mentioned [1]. According to the cancer registry, the incidence of prostate cancer peaks at the age of 70 years in the general population [27], which may explain the high rate of prostate cancer in the highest groups of all exposure metrics in the present study, despite the adjustment for age in the multivariate analyses.

As in the study of the Nordic pilots [1], the pilots in the present study are considered to belong to social class I. That is one of the defects when comparing pilots with the general population, as discussed previously [1]. However, the ratios for melanoma and BCC in the present study were much higher than expected, 3.31 and 2.49, respectively. The possible bias due to social class difference may be eliminated in the internal analyses of the present study. The pilots in the present study were all licensed commercial pilots; some had been employed at the only long-term operating airline in the country, while other pilots were known to have never been employed at the airline, according to the airline files. Both groups belong to the same social class, measured in terms of education.

In the previous study of Icelandair pilots [2], an attempt was made to evaluate the possible association between cancer risk and disturbance of the circadian rhythm. In the present enlarged cohort, it was not possible to differentiate between pilots who had ever flown to North America and those who had only flown within Europe, as all Icelandair pilots had now flown alternately eastward and westward routes from Iceland. So it was not possible to include jet lag factor into the multivariate analysis. The time difference between Iceland and the east coast of North America (Eastern Time) is 4 to 5 h, and the difference between Iceland and mainland Europe is 0 to 2 h. According to the flight schedules, the pilots had at maximum crossed five time zones in a leg. Thus there was no huge difference and jet lag is not considered as a serious confounder in the internal analyses in present study.

Studies on the nature of the interaction between ultra violet radiation (UVR) and ionizing radiation on the risk of BCC have not been conclusive [16, 21]. A questionnaire study of commercial pilots indicates association of childhood sunburns, flight time at high latitude, and family history of non-melanoma skin cancer [28]; however these studies support multi-factorial causes of BCC.

Studies on the transmittance of UVR of aircraft windscreens are in agreement that UV-B is almost completely blocked [29–31] and this part of the UVR spectrum is considered the most potent in terms of carcinogenicity [32]. The glass laminated windscreen transmittance of UV-A in commercial aircraft was approximately 50% at the mid-wavelength of the UV-A bandwidths [30]. This part of the UVR spectrum is considered carcinogenic to humans based on mechanistic data only, as human studies were lacking [32], and the UVA-radiation is the subtlest form of UVR in terms of possible carcinogenicity. It has been postulated that the increase in melanoma among pilots could be the result of penetration of the UV-A radiation though the windshield of the cockpit and in these speculations one has also to assume that the UV-A radiation passes through the uniforms of the pilots [31], notwithstanding that the windows of the cockpits are small, and that this exposure does not closely match the body location of the melanomas. Neither does this explain the increased incidence of melanoma in cabin crew, some of whom are employed at the same airlines [8]. The authors of these speculations on UV-A penetration into the cockpit [31] made some mistakes when selecting the studies to include in their meta-analysis of melanoma in airline pilots and cabin crew [33]: they failed to include the Nordic study of pilots [1]. Instead they compiled four national studies from Nordic countries [33], and left out the fifth cohort, the Finnish pilots, which was not published separately, but was included in the larger study [1]. In doing so, it seems that they did not notice, or at least they did not comment on, the anatomical distribution of the melanomas, or on the fact that the relative risk of melanoma increased with the estimated ionizing radiation dose [1]. Because of the large size of this joint analysis of the five national cohorts of commercial airline pilots, it was possible to study with confidence the location of the melanomas and the dose-response relationship [1].

### Strength and limitation

The estimates of the cumulative dose of cosmic radiation were based on precise information accumulated at the airline, and using the CARI-6 software [13]. The estimates obtained from CARI-6 software correlate with empirical measurements [15]. The long follow-up time strengthens the study. We used the comprehensive population registries and the personal identification number in an accurate record linkage. That procedure enabled obtaining date of eventual out-migration, and date of death from the National Registry; and the date of diagnosis of cancer cases from the Cancer Registry. The Cancer Registry has virtually complete coverage and over 95% of the diagnoses are histologically confirmed [27, 34]. The use of cancer incidence in the present study is a clear advantage as compared with the use of mortality data in the evaluation of cancer risk among pilots because the cancer types involved are those with good prognosis and high survival rate. According to the Cancer Registry, the five-year survivals of malignant melanoma and prostate cancer have been over 80% during the last 25 years [27].

Lack of knowledge of individual exposure to leisure time sun exposure and host factors is a limitation. According to previous study on host factors and leisure sun-exposure, there was no substantial difference between pilots and the general male population in Iceland [12], rendering confounding due to these factors unlikely concerning skin cancers.

Despite having long follow-up, the small size of the study is an obvious handicap, leaving us with wide 95% CIs, and few cases at rare cancer sites, albeit in some cases the 95% CIs did not include unity. These are shown here for completeness, and for future meta-analyses. This precluded certain analysis of brain, thyroid, squamous cell skin cancers, and leukaemia, which have been the subject of other studies on pilots. Information on host factors for skin cancer and leisure sun-exposure were not available for adjustment in the multivariate analyses in the present study; however, confounding due to these risk factors for skin cancers is unlikely compare previous study where these risk factors were reported to be equally distributed between pilots and the population [12]. Similarly, possible disturbance of circadian rhythm is not controlled for; however, it may be of minor importance, as the pilots crossed five time zones at the most. Another study of aircrew has shown that circadian rhythm disruption due to crossing many time zones was strongly correlated with cumulative cosmic radiation dose [35], so these exposures are difficult to disentangle.

The possibility of detection bias may arise if access to health services across populations is different in a comparative study. Pilots, of high social class, are partially selected prior to licence on account of their health and physical fitness, and undergo continuing medical check-ups, and have thus easy access to medical doctors. This may cause bias, particularly in the comparison with the general population, but this is not as probable in the internal analyses. A clear healthy worker effect was not observed in the present study. Earlier mortality study of the cohort showed increased standardized mortality ratio for melanoma and prostate cancer, albeit not statistically significant [36], and that does not support the suggestion that these cancers were over-diagnosed in the present study. No systematic screening with prostate-specific antigen has ever been implemented for the general population in Iceland, and it is not known whether or to what extent the pilots or the general male populations have been tested by prostate-specific antigen.

## Conclusions

In conclusion, the results of this study of licenced commercial pilots show a similar pattern to that reported in previous cancer incidence studies, namely higher incidence of malignant melanoma, and BCC compared with the general population. In addition, the risk for all cancers, melanoma, prostate cancer, BCC, BCC of trunk increased with an increase in the exposure metrics: number of employment years, cumulative air hours, total cumulative radiation dose, and cumulative radiation dose up to the age of 40 years in an exposure-response manner of relationship. BCC is radiation-related cancer, and may be attributed to cosmic radiation. Confounding is unlikely due to leisure sun exposure and host factors associated with skin cancer as these were previously reported of similar frequency among pilots and among the general population. Further studies are needed to exactly clarify the risk of cancers in association with cosmic radiation. These should aim at control of other workplace exposure, and lifestyle factors, as clothes, and glass in small cockpit windows shield pilots from the most potent ultraviolet-radiation.

## Abbreviations

BCC: Basal cell carcinoma of skin
CI: Confidence interval
CT: Computed tomography
IARC: International Agency for Research on Cancer
mSv: MilliSievert
RR: Relative risk
SIR: Standardized incidence ratio
UV-A: Ultraviolet a-band
UV-B: Ultraviolet b-band
UVR: Ultraviolet radiation
